# First laboratory-confirmed case of scrub typhus in Shijiazhuang City, Hebei Province

**DOI:** 10.3389/fmicb.2024.1409949

**Published:** 2024-05-24

**Authors:** Huixiu Lu, Jianying Li, Rong Fan, Gaoyuan Hao, Meilan Sun, Yunchuan Liang

**Affiliations:** ^1^Department of Dermatology, Shijiazhuang People’s Hospital, Shijiazhuang City, Hebei, China; ^2^Department of Laboratory Medicine, Shijiazhuang People’s Hospital, Shijiazhuang City, Hebei, China

**Keywords:** scrub typhus, *O. tsutsugamushi*, Kawasaki genotype, phylogenetic analysis, indirect immunofluorescence assay

## Abstract

**Objective:**

Defining whether a suspected case was due to scrub typhus through laboratory testing, to understand the prevalence of scrub typhus in Shijiazhuang City, Hebei Province.

**Methods:**

An epidemiological investigation was conducted on the suspected case, utilizing Weil-Felix test and indirect immunofluorescence assay (IFA) to detect specific antibodies against *O. tsutsugamushi* in serum specimens. Additionally, PCR amplification of the 56-kDa and *groEL* genes was performed, followed by constructing a phylogenetic tree to identify the genotype.

**Results:**

The acute phase titer of the Weil-Felix test for the case was 1:160, which increased to 1:320 in the recovery phase. IFA assay revealed IgG titers against *O. tsutsugamushi* of 1:64 in the acute phase and 1:256 in the recovery phase. Sequence alignment of the PCR amplified fragment showed the highest similarity with the *O. tsutsugamushi* genotype. Kawasaki sequence, ranging from 99.71 to 100.00%. The strain exhibited the closest genetic relationship with the known *O. tsutsugamushi* Kawasaki genotype.

**Conclusion:**

This study confirms the presence of *O. tsutsugamushi* in Shijiazhuang City, Hebei Province, with the identified strain belonging to the Kawasaki genotype, marking the first diagnosis of this strain in the region.

## 1 Introduction

Tsutsugamushi disease, also known as scrub typhus, is transmitted by chiggers. Humans typically contract this acute infectious disease through bites (Tamura et al., 1995). It was previously believed to only involve one species, *O. tsutsugamushi*, and was confined to the area designated as the “tsutsugamushi triangle”, which extends from Afghanistan through China and Korea, covering the western Pacific and Indian Ocean islands, and reaching northern Australia. Around one billion individuals face the risk of scrub typhus (Kelly et al., 2009), with approximately one million cases reported each year (Watt and Parola, 2003; Hu et al., 2015). However, recently, cases have been reported in several regions of South America and Chile, where a new pathogen, cand. *O. chiloensis*, has been discovered (Abarca et al., 2020; Jiang et al., 2022). Additionally, pathogens such as cand. *O.chuto* have been found in the United Arab Emirates and Kenya (Izzard et al., 2010; Masakhwe et al., 2018). This indicates that the risk of scrub typhus caused by different species of Orientia is broader and more significant than previously known, potentially having global implications.

Before 1986, reports and outbreaks of scrub typhus cases were mainly concentrated in regions south of the Changjiang River in China (Yu, 1997). Subsequently, the epidemic continuously spread and expanded to northern regions of the country. In the north, strains with lower virulence such as Gilliam, Kawasaki, and Yonchon genotypes, were predominated (Zhang et al., 2014). In 1997, the presence of scrub typhus was first reported in the Taihang Mountain area of Hebei Province (Chen et al., 2000). However, due to Hebei being in a transitional endemic area with strains of lower virulence, there have been few documented cases and related investigation (Lu et al., 2018).

This study conducted laboratory diagnosis of a scrub typhus case in Shijiazhuang City, Hebei Province. Utilizing serological and molecular biology methods, we confirmed that the case was caused by the Kawasaki strain. This research aims to elucidate the genotype of *O. tsutsugamushi* in Hebei Province, provide theoretical basis and reference for clinical diagnosis.

## 2 Materials and methods

### 2.1 Sample collection

**FIGURE 1:**
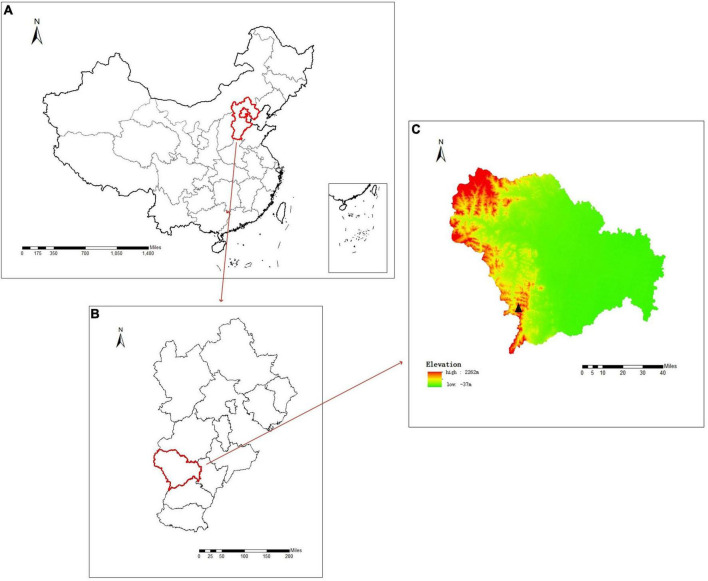
Map of the study area. **(A)** Map of China. **(B)** Map of Hebei Province. **(C)** Map of Shijiazhuang City, Hebei Province, Black triangle marks location where patient went mountain climbing, Jingxing County, Shijiazhuang City, Hebei Province, China.

On October 13, 2023, a case of scrub typhus was diagnosed at the People’s Hospital of Shijiazhuang City (Figure 1). Blood samples of 5 ml were collected from the patient on the 13th and 21st of the same month for further analysis. Subsequently, 3 ml of the blood specimen was processed for serum isolation, and the isolated serum was cryopreserved at −20°C. Simultaneously, the remaining 2 ml of blood underwent DNA extraction following the standard protocol outlined in a QIAamp DNA Blood Mini Kit (Qiagen, Germany), and was stored at −20°C until use.

### 2.2 Weil-Felix test

Serological evaluation of OXK, OX19, and OX2 antibodies in patient serum was conducted using a commercial testing kit from Senson Reagent Co., Ltd. (Shanghai, China). The testing procedures strictly followed the standard operating protocol provided by the kit manufacturer and complied with relevant regulations outlined in the “National Clinical Laboratory Procedures” (Shang et al., 2015).

### 2.3 Indirect immunofluorescent antibody method

Patient’s sera were tested for serum-specific IgG antibodies against *O. tsutsugamushi* using commercial immunofluorescence assay (IFA) kits (Scimedx, USA). The testing procedures were conducted following the standard operating protocols provided by the kit manufacturer.

### 2.4 Nucleic acid amplification and detection

Amplification of the 56-kDa and *groEL* target genes in the samples using the nested PCR method, with the primer sequences required for each amplification listed in Table 1. The amplicons were isolated with the QIAquick PCR Purification Kit (Qiagen, Hilden, Germany) and sent to Beijing Tianyi Huiyuan Biotechnology Company (Beijing, China) for sequencing. The agarose gel electrophoresis images are provided in the Supplementary Data.

**TABLE 1:** Primers used for amplification of marked genes of *O. tsutsugamushi.*

Gene	Primer name	Sequences (5′→3′)	Anneal temperature	Length of coding sequence	References
56-kDa	P34-F1	TCAAGCTTATTGCTAGTGCAATGTCTGC	55°C	_	Furuya et al., 1993
	P55-R1	AGGGATCCCTGCTGCTGTGCTTGCTGCG			
	P10-F2	GATCAAGCTTCCTCAGCCTACTATAATGCC	55°C	485 bp	
	P11-R2	CTAGGGATCCCGACAGATGCACTATTAGGC			
*groEL*	Gro-F1	AAGAAGGA/CGTGATAAC	50°C	_	Chen et al., 2000
	Gro-R1	ACTTCA/CGTAGCACC			
	Gro-F2	ATATATCACAGTACTTTGCAAC	50°C	365 bp	
	Gro-R2	GTTCCTAACTTAGATGTATCAT			

### 2.5 Phylogenetic data analysis

The obtained DNA sequences were compared to those available in GenBank using BLAST.^1^ The sequences reported in this paper have been deposited in GenBank with the accession numbers for genes of 56-kDa (PP504740) and *groEL* (PP504739). Phylogenetic and molecular evolutionary analysis was conducted using the neighbor-joining method with 1000 replicates for bootstrap analysis in MEGA 7.0.

## 3 Results

### 3.1 Case presentation

**FIGURE 2:**
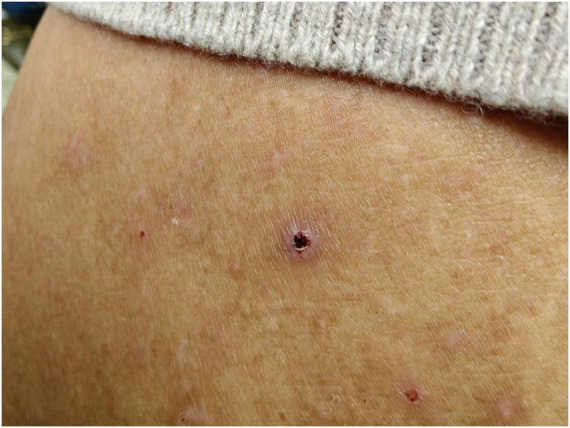
Images of scab on the left side of the patient abdomen.

A 47-year-old male urban resident, who had taken his pet dog mountain climbing at Jingxing County, Shijiazhuang, Hebei Province (Figure 1) a week before, presented with persistent fever and subsequently self-administered Cefalexin Tablets. During this visit, his body temperature was recorded as 38.1°C, blood pressure was 130/90 mmHg, and auscultation revealed normal sounds. His heart rate was 70 beats per minute, regular, with no murmurs detected. The patient exhibited symptoms including sore throat, decreased appetite, chest tightness, and fatigue. Physical examination revealed ulcerations at the site of insect bites, characterized by scabs with a central depression surrounded by whitish-red circular areas, approximately the size of a mung bean. There was no associated pain or itching. The scab was located on the left side of the abdomen (Figure 2). A blood routine examination showed an elevated neutrophil count and decreased lymphocyte and platelet counts. The patient was treated with doxycycline, orally, 0.2 g per dose, once daily for 7 days. After the treatment, there was a significant improvement in the patient’s condition 2 days the treatment, with normalization of body temperature, relief of headache.

### 3.2 Weil-Felix test

After 20 h of incubation, the acute phase serum sample showed agglutination at a 1:160 dilution for OXk antibody detection, while the convalescent phase sample exhibited agglutination at a 1:320 dilution. Furthermore, the agglutination titer for Proteus OX19 and OX2 was below 1:80. According to the diagnostic criteria, a agglutination titer of less than 1:80 for OX19 and OX2 excludes the possibility of rickettsial infection, while an OXk agglutination titer of 1:160 or higher is consistent with the diagnosis of scrub typhus infection.

### 3.3 Indirect immunofluorescent antibody method

**FIGURE 3:**
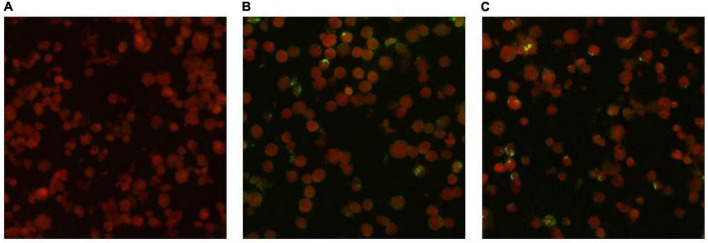
Detection results of anti-O. tsutsugamushi IgG in serum by IFA method. **(A)** Negative control. **(B)** Acute phase titer: 1:64. **(C)** Convalescent phase titer: 1:256.

IgG antibodies for *O. tsutsugamushi* were positive in both serum samples. The acute phase serum sample showed an IgG antibody titer of 1:64, while the convalescent phase serum sample showed an IgG antibody titer of 1:256. The antibody titer in the convalescent serum was four times higher than that in the acute phase serum, meeting the diagnostic criteria for Scrub typhus infection. The results of the serum antibody tests are shown in Figure 3.

### 3.4 Sequencing and phylogenetic analysis

**FIGURE 4:**
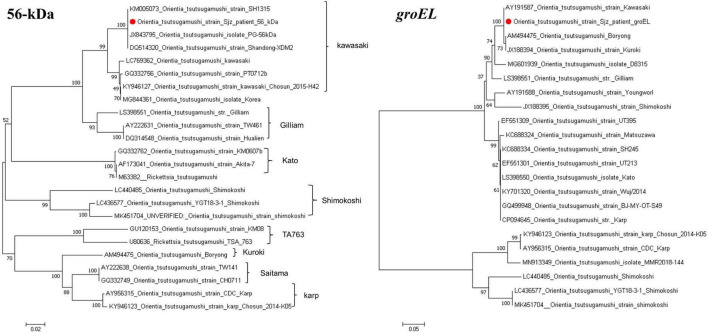
Phylogenetic trees based on the nucleotide sequences of 56-kDa and *groEL* genes from *O. tsutsugamushi* strains. Sequences obtained in this study are marked with a red dot before their names.

Using the primers listed in Table 1, targeted gene PCR amplification was performed on blood samples. The amplified gene fragments were named *O. tsutsamushi* Sjz patient. These fragments were subsequently results were compared and analyzed with known sequences in GenBank. In the homology analysis of the 56-kDa gene, the *O. tsutsugamushi* Sjz patient strain detected in this study showed a homology of 99.69–99.85% with known strains of *O. tsutsugamushi* Kawasaki genotype, including SH1315 (KM005073), PG (JX843795), and Shangdong-XDM2 (DQ514320). In the homology analysis of the *groEL* gene, the *O. tsutsugamushi* Sjz patient strain exhibited homology of 99.71–100.00% with Kawasaki (AY191587), Boryong (AM494475), and Kuroki (JX188394) strains. Evolutionary trees were constructed using the 56-kDa and *groEL* genes, showing that the *O. tsutsugamushi* Sjz patient strain clustered with known Kawasaki genotype strains, indicating that the strain infecting this case is of the same genotype as *O. tsutsugamushi* Kawasaki (Figure 4).

## 4 Discussion

Hebei Province is situated in the intermediary zone between the northern and southern endemic regions. The province showcases a variety of terrains, encompassing undulating hills, forests, and plains. Its Taihang Mountain area was designated as a natural focus of Scrub typhus as early as 1997, with the initial confirmed case of Scrub typhus in Hebei Province emerging from this locality (Zhang et al., 2013; Li et al., 2020). In 2018, Hebei Province confirmed its first case of Kawasaki disease caused by *O. tsutsugamushi* infection, located in Baoding City, Hebei Province (Lu et al., 2018). The genotype of this case matched that of cases in Shandong in 2013 and showed a certain trend of transmission (Li et al., 2013). Serving as the provincial capital of Hebei, Shijiazhuang falls under the category of a transitional endemic region, located in the central-southern part of the province characterized primarily by flat topography. The cityhas no documented Scrub typhus so far. This research presents a laboratory-confirmed case of Scrub typhus in Shijiazhuang, marking the city’s inaugural instance.

The case indicates that the patient has a history of outdoor exposure, presenting with a fever and a specific scab. *Orientia spp.* specific PCR methods targeted 56-kDa and *groEL* genes were positive and phylogenetic analysis showed the causative agent was closed to Kawasaki genotype. The Weil-Felix test shows OXk antibody titer above 1:80. Serum antibody testing shows positive IgG antibodies to *O. tsutsugamushi* in both acute and convalescent phase samples, with the IgG antibody titer in convalescent phase samples being four times higher than that in the acute phase. Consistent with the clinical diagnostic criteria for scrub typhus infection (Yang and Ren, 2012; Lu et al., 2023). These results indicate that the patient is suffering from scrub typhus.

Hospitals commonly rely on the Weil-Felix test for scrub typhus diagnosis due to its simplicity and ease of use, but it has limitations in sensitivity and specificity, leading to potential misdiagnosis (Sadanandane et al., 2018). Although IFA is considered the gold standard, its diagnostic value is hindered by subjectivity and antigen restrictions. In contrast, molecular biology techniques offer advantages in sensitivity, specificity, speed and simplicity, and are widely used in identifying and typing *O. tsutsugamushi*, the causative agent of scrub typhus.

*O. tsutsugamushi* infection exhibits diversity. Homology analysis of the 56-kDa antigen gene sequence indicates approximately nine genotypes: Karp, Kato, Gilliam, Kawasaki, Kuroki, Saitama, TA763, JG, and Shimokoshi (Kelly et al., 2009; Lu et al., 2010). Based on varying virulence in mice, strains are classified as highly virulent, moderately virulent, or weakly virulent, with Karp and Kato classified as highly virulent, Kawasaki and Kuroki as weakly virulent, and Gilliam exhibiting intermediate virulence. Gene sequencing and phylogenetic analysis revealed high similarity of the patient’s *O. tsutsugamushi* strain 56-kDa and *groEL* genes to the known Kawasaki genotype, providing strong evidence of infection with an *O. tsutsugamushi* Kawasaki genotype strain in this case.

This study identified a patient with a scab in Shijiazhuang City, Hebei Province. Through epidemiological investigation, clinical diagnosis, and laboratory testing, the patient was ultimately diagnosed with Scrub typhus infection. This represents the first laboratory confirmed case of Scrub typhus in Shijiazhuang City, filling a gap in reported cases and enriching the pathogen spectrum of *O. tsutsugamushi* infections in Hebei Province. This discovery provides important assistance for the diagnosis of scrub typhus in hospitals in the region and establishes clinical evidence. The report raises awareness among healthcare professionals about the presence of scrub typhus in Shijiazhuang City and contributes to the development of effective diagnostic and preventive measures.

The study is subject to limitations, primarily due to the inability to definitively determine the transmission route of the disease. Despite detailed analysis of the cases, the lack of sufficient data on vectors and host animals precludes the establishment of its transmission mechanism. Additionally, reliance solely on two gene segments for the identification of the spotted fever strain through genetic sequence analysis may introduce inherent limitations (Batty et al., 2018). Future research efforts could address these constraints through comprehensive epidemiological investigations and whole-genome sequencing, ensuring the comprehensiveness and accuracy of identification results.

## Data availability statement

The datasets presented in this study can be found in online repositories. The names of the repository/repositories and accession number(s) can be found in the article/Supplementary material.

## Ethics statement

The studies involving humans were approved by the Ethics Committee of the First People’s Hospital of Shijiazhuang City (No. 201706). The studies were conducted in accordance with the local legislation and institutional requirements. The participants provided their written informed consent to participate in this study. Written informed consent was obtained from the individual(s) for the publication of any potentially identifiable images or data included in this article.

## Author contributions

HL: Conceptualization, Methodology, Writing – original draft, Writing – review and editing. JL: Methodology, Writing – original draft. RF: Writing – original draft. GH: Writing – original draft. MS: Writing – original draft. YL: Writing – review and editing.

## Footnotes

1
https://blast.ncbi.nlm.nih.gov/Blast.cgi
