# Supplementary material for: First laboratory-confirmed case of scrub typhus in Shijiazhuang City, Hebei Province
Source: Front Microbiol. 2024 May 24;15:1409949. doi: 10.3389/fmicb.2024.1409949 (PMC11157097; doi:10.3389/fmicb.2024.1409949)
Supplement: Supplementary file 1 [file Data_Sheet_1.docx]

Supplementary Material

# Supplementary Data

The electrophoretic detection of ampliﬁcation PCR products on agarose gel.


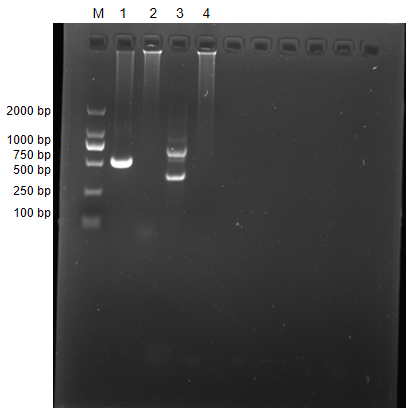


Electrophoretical detection of amplification of *O. tsutsugamushi* on agarose gel. M: 2000 bp marker; line 1: 56-kDa genes; line 2: Negative control for 56-kDa; line 3: *groEL* genes; line 10-line 12: Negative control for *groEL* genes.
